# Evaluation of Suprahyoid Muscle Thickness, Swallowing Anxiety, and Water-Swallowing Performance in Healthy People with Pill Dysphagia: A Cross-Sectional Study

**DOI:** 10.3390/healthcare14193167

**Published:** 2026-09-24

**Authors:** Birce Zorlutuna, Cetin Sayaca, Ozlem Turkoglu

**Affiliations:** 1 Department of Physiotherapy and Rehabilitation, Faculty of Health Sciences, Uskudar University, İstanbul 34768, Türkiye; bir.cee94@gmail.com; 2 Department of Physiotherapy and Rehabilitation, Faculty of Health Sciences, Bursa Uludag University, Bursa 16059, Türkiye; 3 Department of Radiology, Faculty of Medicine, University of Health Sciences, İstanbul 34668, Türkiye; ozlemkolcak@hotmail.com

**Keywords:** suprahyoid muscles, thickness, pill dysphagia, water-swallowing performance, anxiety

## Abstract

Background: Pill dysphagia (PD) is a serious problem affecting healthy individuals, leading to poor medication adherence and potential health risks. The relationship between suprahyoid muscle thickness and swallowing performance remains underexplored in PD. This study aimed to evaluate swallowing anxiety, water-swallowing performance with pills, and the thickness of the suprahyoid muscles of healthy individuals with PD. Methods: Swallowing anxiety, water-swallowing performance with pills, muscle thickness on B-ultrasound, and the level of difficulty felt during pill swallowing were evaluated in healthy individuals with and without PD. Results: The study included 20 subjects with PD (PD group) and 20 subjects without PD (control group), comprising 27 males and 13 females with a mean age of 23.98 ± 2.97 years (18–32 years). The thickness values of the anterior venter of the digastric muscle, the mylohyoid, and geniohyoid muscles were greater in the control group than in the PD group (*p* < 0.000, *p* < 0.000, *p*:0.007, respectively). The severity of swallowing difficulty, swallowing anxiety, and the degree of difficulty experienced when swallowing pills were determined to be higher in the PD group (*p*:0.001, *p*:0.001, *p* < 0.000, respectively). Water-swallowing speed and capacity with/without pills were better in the control group (*p* < 0.000; *p* < 0.000; *p*:0.005; *p* < 0.000, respectively). No difference was observed between the groups in the volume of water swallowed with and without pills (*p*:0.468, *p*:0.162, respectively). Conclusions: In healthy individuals with pill dysphagia, a reduction in the thickness of the suprahyoid (SH) muscles may be associated with pill dysphagia. Randomized controlled trials investigating the effects of SH muscle exercises in healthy individuals with dysphagia are needed.

## 1. Introduction

Pills are the most common, preferred, and convenient form of medication administration [1]. Pill dysphagia (PD) is defined as one of the swallowing disorders seen in clinical practice. The frequency of PD increases with aging and is a common and costly problem [2]. Individuals with dysphagia are known to experience difficulties swallowing pills [3]. In the literature and clinical practice, this condition is considered normal in older adults and/or individuals with swallowing difficulties. Contrary to popular belief, it is stated that PD occurs in approximately 40% of healthy individuals [4]. It has been reported that 30–40% of children refuse to swallow medication, and 50% cannot swallow standard and/or small-size pills [5]. It has also been determined that >30% of young, healthy individuals experience PD for reasons such as taste, size, sensation, or physical discomfort [6].

PD can cause a decrease in the bio-benefits and pharmacokinetic efficacy of the drug due to pill retention injuries [7,8,9], aspiration [10,11], and poor drug compliance [12,13,14]. This affects treatment adherence, increasing morbidity and mortality risks [15]. It has been reported that 14% of individuals with PD delay taking a dose of the drug, and 8% completely avoid taking the drug [4]. Approximately 10% of patients going to community pharmacies are known to modify their medications because of PD [16], and up to one-third of these modifications are inappropriate [17]. Changing pills to be able to swallow them more easily can be dangerous and lead to medication intake issues, choking, and even death [18]. In the literature, PD may be due to oropharyngeal sensitivity and motor defects [4], esophageal narrowing, web formation, motility disorders [19], or phagophobia [20].

To overcome the problems arising from PD and develop effective strategies, research into the underlying causes of PD is needed [21]. Physiological changes, such as dysfunction in the muscles responsible for swallowing, have been shown to increase the risk of swallowing difficulties [22]. No study could be found in the literature examining water-swallowing performance with pills and suprahyoid (SH) muscle thickness in healthy individuals who have PD. The present study aimed to compare swallowing anxiety, the thickness of the SH muscle, and water-swallowing performance with pills in healthy individuals with and without PD. Our hypothesis was that “Healthy individuals with PD-related swallowing anxiety, SH muscle thickness, and water-swallowing performance with pills differ from those of healthy individuals without PD”.

## 2. Materials and Methods

### 2.1. Study Design and Population

This study was designed as a cross-sectional study. The present study was conducted at the Swallowing Disorders Unit of the Üsküdar University Physical Therapy and Rehabilitation Application and Research Center (ÜSFİYOTEM). The Non-invasive Clinical Research Ethics Committee approved the study protocol (Decision No: 61351342/2020-164). All patients were informed of the scope and procedures of the study. Written informed consent forms were signed by all participants before the study, and the study was conducted in accordance with the ethical guidelines and principles of the Declaration of Helsinki.

In the sample size analysis, considering an alpha significance level of 0.05, a power value of 0.80, an effect size of 0.80, and equal numbers of individuals in each group, it was calculated that a total of 42 individuals were required, with at least 21 participants per group. These calculations were performed using the G*Power 3.1.9.2 software (Heinrich Heine University Düsseldorf, Düsseldorf, Germany).

Participants in the pill dysphagia (PD) group were recruited from individuals who were directly admitted to our clinic with complaints of PD, as well as through invitations distributed via online professional and social media networks. Healthy individuals with PD were included in the PD group, whereas healthy individuals without any complaints of PD were included in the control group. To ensure a robust study sample, the PD group specifically consisted of individuals aged 18–40 years who had a complaint of PD and had previously undergone formal medical examinations by relevant specialists (e.g., otolaryngologists, neurologists, or gastroenterologists) to investigate this specific complaint, ensuring that no participants with unexamined or new-onset symptoms were included. Furthermore, all subjects in the PD group scored ≥ 2 points on the Eating Assessment Tool-10 (EAT-10). The control group consisted of individuals aged 18–40 years with no complaints of PD who scored 0 points on the EAT-10, were selected randomly from the volunteer pool, and who met the inclusion criteria to form a comparable cohort. Clinical swallowing evaluations of all participants were performed by a senior specialist with at least 10 years of clinical experience in swallowing disorders. Ultrasonographic (US) examinations of the suprahyoid muscles were conducted by an expert radiologist specializing in musculoskeletal imaging. Participants in both the patient and control groups were excluded from the study if they presented with any active neurological disease, a history of aspiration pneumonia, any gastroesophageal disease (e.g., reflux, cancer), current or past orthodontic treatment, a history of swallowing rehabilitation, or concurrent psychological treatment.

### 2.2. Evaluation

The demographic information, as well as the assessments of the Severity of Swallowing Disorder, Swallowing Anxiety, and the Degree of Difficulty in Swallowing Pills, were first performed and kept confidential from the experts conducting the other assessments. The Water-Swallowing Performance assessment was conducted by a physical therapist with extensive experience in swallowing disorders. The thickness of the SH muscle was assessed by a radiologist specializing in this field. Neither the radiologist nor the physical therapist knew which group the individuals belonged to.

**Severity of Swallowing Disorder (SD):** The Turkish version of the EAT-10 Questionnaire was used to determine SD [23]. This instrument consists of ten questions, each scored between ‘0’ (no problem) and ‘4’ (severe problem). The total score ranges from 0 to 40 points. A total score of 2 or more points can indicate swallowing dysfunction [24]. The EAT-10 score was used as an inclusion criterion in the present study.

**Swallowing Anxiety (SA):** SA was assessed using the Swallowing Anxiety Scale (SAS), which has been proven to be a valid and reliable measurement method for the Turkish population. The scale consists of 10 questions, each scored from 0 to 4 points, for a maximum score of 40 points. Higher scores indicate increased swallowing anxiety. Swallowing anxiety is a psychological concept that describes a functional swallowing disorder [25].

**Thickness of the SH Muscle:** SH thickness in the study participants was evaluated using ultrasonography (USG). The study participants were given detailed information before the USG evaluation. All USG measurements were taken using a Siemens Acuson S3000 diagnostic ultrasound system (Siemens Medical Solutions USA, Inc., Issaquah, WA, USA). In assessing muscle thickness with USG, the individual was positioned sitting upright and asked to keep their mouth closed with the jaw relaxed. The USG probe was placed below the jaw in a coronal position, perpendicular to the jaw at 90°, with a generous amount of contact gel to avoid excessive pressure on the skin. Muscle thickness was measured with electronic calipers at standard locations. The digastric muscle was measured from the upper to the lower boundary of the fascia at the broadest point perpendicular to the mylohyoid muscle. The mylohyoid muscle was measured from under the measurement point of the digastric muscle. The geniohyoid muscle was measured from the raphe of the mylohyoid muscle to the boundary of the tongue. All thicknesses of muscles were recorded in millimeters (mm). The researcher evaluating SH thickness was blinded to the study participant groups [26].

**Water-Swallowing Performance:** Swallowing performance was evaluated using the 100 mL water-swallowing test (WST), noted for its affordability, ease of use, and high reliability. Furthermore, it enables regular, frequent monitoring of patients over time [27]. The high intra- and inter-rater reliability and test–retest reliability of water-swallowing speed (mL/s) have been demonstrated [28]. There is a well-established relationship between water-swallowing speed and swallowing difficulty [29]. A total of 100 mL of water was placed in a plastic cup, and the subject was instructed to drink it “as quickly as comfortably possible” while in an upright seated position. The time spent drinking and the amount of liquid swallowed were recorded. If the patient could not swallow all the water, any remaining liquid was measured with a syringe to the nearest 1 mL. The researcher simultaneously counted the number of swallows by sensing laryngeal elevation during swallowing (perceiving the thyroid cartilage). Timing began when the water first touched the lower lip after the “start” command and stopped when the larynx returned to rest after the last swallow (typically accompanied by other signs such as exhalation, phonation, or mouth opening). Three swallowing performance parameters were calculated: swallow volume (ml per swallow = total ml swallowed divided by number of swallows), swallow capacity (ml per second = total ml swallowed divided by time taken), and swallow speed (seconds per swallow = time taken divided by number of swallows) [28]. Patients who completed the 100 mL water-swallowing test were asked to repeat the test using a cellulose placebo capsule (250 mg, measuring 38 × 38 × 70 mm) to evaluate the effect of pill-swallowing on swallowing function. A 5 min break was given for rest between the 100 mL water-swallowing tests with/without pills. The evaluation was conducted using the same brand of bottled water under consistent room conditions to mitigate potential positive or negative influences, although insufficient data were available. The researcher who evaluated the water-swallowing performance with/without pills was blinded to the groups of participants.

**Degree of Difficulty in Swallowing Pills:** A Visual Analog Scale (VAS) was used to assess the difficulty of swallowing pills experienced by study participants. This scale is used to convert values that cannot be measured quantitatively to a numerical form [30]. The subject is instructed to mark the degree of difficulty felt when swallowing a pill on a straight line marked from 0 at one end to 10 at the other. The point indicated on the line was recorded in mm.

### 2.3. Statistical Analysis

The statistical analysis of the data obtained from the study was performed using the “Statistical Package for Social Sciences (SPSS) Version 25.0 (SPSS Inc., Chicago, IL, USA)” software. The conformity of numerical data to a normal distribution was evaluated with the Kolmogorov–Smirnov test. In comparisons between two groups of numerical data, the Independent Samples *t*-test was applied to normally distributed parameters, and the Mann–Whitney U-test was used when the distribution was not normal. The Chi-square test was used to compare qualitative data. A one-way analysis of covariance (ANCOVA) was performed to determine whether there were differences between the groups regarding the muscle thickness of the anterior belly of the digastric, mylohyoid, and geniohyoid muscles, while controlling for the subjects’ sex and BMI. Results were stated with a 95% confidence interval, and a value of *p* < 0.05 was accepted as the level of statistical significance.

## 3. Results

The evaluation was conducted on 40 individuals (27 females and 13 males) with a mean age of 23.98 ± 2.97 years. The demographic information (age, gender, height, weight, and body mass index [BMI]) of the participants is presented in Table 1. No significant differences were observed between the groups in age, height, weight, and BMI (*p* > 0.05).

The results of the comparisons of individuals with and without PD with respect to SH thickness, water-swallowing performance with/without pills, the severity of swallowing disorder, the level of swallowing anxiety, and the degree of difficulty experienced when swallowing pills are presented in Table 2. Significant differences were determined between the PD group and the control group in the thickness values of the anterior venter of the digastric muscle, mylohyoid muscle, and geniohyoid muscle (*p* < 0.000; *p* < 0.000; *p*:0.007, respectively). The thickness values of the anterior venter of the digastric, mylohyoid, and geniohyoid muscles were higher in the healthy control group.

Statistically significant differences were observed between the two groups with respect to the severity of swallowing difficulty, swallowing anxiety, and the degree of difficulty experienced when swallowing pills (*p*:0.001; *p*:0.001; *p* < 0.000, respectively). Individuals with PD had greater severity of swallowing difficulty, higher levels of swallowing anxiety, and experienced greater difficulty when swallowing pills. According to the results of the WST with/without pills, there were significant differences between the groups regarding the speed of swallowing water without pills, the water-swallowing capacity without pills, the water-swallowing speed with pills, and the water-swallowing capacity with pills (*p*:0.005; *p* < 0.000; *p* < 0.000; *p* < 0.000, respectively). The water-swallowing speed and capacity were better in the healthy control group than in the PD group, both with/without pills. No difference was determined between the groups with respect to water-swallowing volume with/without pills (*p*:0.468; *p*:0.162, respectively) (Table 2).

A one-way analysis of covariance (ANCOVA) was conducted to determine whether there were significant differences in the thickness of the digastric (anterior venter), mylohyoid, and geniohyoid muscles between the groups while controlling for the individuals’ sex characteristics. The effect of sex, included in the model as a covariate, was found to provide significant control over digastric muscle thickness (F(1,37) = 16.30, *p* < 0.001, η^2^ = 0.31), mylohyoid muscle thickness (F(1,37) = 9.75, *p* = 0.003, η^2^ = 0.21), and geniohyoid muscle thickness (F(1,37) = 11.13, *p* = 0.002, η^2^ = 0.23). These findings indicate that sex differences alone accounted for 31% of the variance in digastric muscle thickness, 21% of the variance in mylohyoid muscle thickness, and 23% of the variance in geniohyoid muscle thickness. When the adjusted mean values according to the sex factor were examined, the adjusted mean digastric muscle thickness of the individuals in the control group (Mean_adj_= 7.96 mm; Std. Error = 0.22 mm) was found to be significantly higher than that of the individuals with difficulty swallowing pills (Mean_adj_ = 6.86 mm; SE = 0.22 mm). The adjusted mean mylohyoid muscle thickness of the individuals in the control group (Mean_adj_ = 20.39 mm; Std. Error = 0.53 mm) was found to be significantly higher than that of the individuals with difficulty swallowing pills (Mean_adj_ = 17.13 mm; SE = 0.53 mm). Additionally, the adjusted mean geniohyoid muscle thickness of the individuals in the control group (Mean_adj_ = 8.98 mm; Std. Error = 0.29 mm) was found to be significantly higher than that of the individuals with difficulty swallowing pills (Mean_adj_ = 8.08 mm; SE = 0.29 mm). A one-way analysis of covariance (ANCOVA) was also applied to determine whether there was a significant difference in suprahyoid muscle thicknesses (digastric, mylohyoid, and geniohyoid) between healthy individuals with and without difficulty swallowing pills while controlling for the individuals’ mean BMI. The effect of BMI, included in the model as a covariate, was found to provide a statistically significant control over digastric [F(1,37) = 5.73, *p* = 0.022, η^2^ = 0.13] and geniohyoid [F(1,37) = 7.43, *p* = 0.010, η^2^ = 0.17] muscle thicknesses; however, it was determined to have no significant effect on mylohyoid muscle thickness [F(1,37) = 3.00, *p* = 0.092, η^2^ = 0.08]. After statistically controlling for the participants’ BMI values, the main effect of the group variable on all muscle thicknesses was found to be significant. Accordingly, a significant difference was found between the groups in digastric muscle thickness [F(1,37) = 11.10, *p* = 0.002, η^2^ = 0.23], showing that the group variable explained 23% of the variance in digastric muscle thickness. Similarly, a highly significant difference between the groups was observed in mylohyoid muscle thickness values [F(1,37) = 17.73, *p* < 0.001, η^2^ = 0.32], and the group effect was found to account for 32% of the total variance (the highest effect size). Finally, the difference between the groups in geniohyoid muscle thickness was also statistically significant [F(1,37) = 4.63, *p* = 0.038, η^2^ = 0.11], and the group effect was found to explain 11% of the total variance. When descriptive statistics and adjusted values were evaluated as a whole, it was concluded that even when the participants’ body mass indexes were controlled, all suprahyoid muscle thickness values of the individuals with normal development (digastric M = 8.14 mm; mylohyoid M = 20.71 mm; geniohyoid M = 9.17 mm) were significantly higher compared to the muscle thickness values of the individuals experiencing difficulty swallowing pills (digastric M = 6.69 mm; mylohyoid M = 16.82 mm; geniohyoid M = 7.90 mm). ANCOVA results for suprahyoid muscle thicknesses by group, controlling for sex and BMI, are given in Table 3. A scatter plot of water swallow volume with pill and mylohyoid thickness by group was shown in Figure 1.

## 4. Discussion

The aim of the present study was to compare swallowing anxiety, the thickness of the SH muscles (anterior ventral section of the digastric muscle, mylohyoid muscle, geniohyoid muscle), and water-swallowing performance with a pill between healthy individuals with and without PD. The first and most important finding of the present study was that all SH muscle thickness values were lower in the PD group than in healthy individuals. A second important finding was that water-swallowing speed with and without a pill was slower, and the amount of water in each swallow was less in the individuals with PD than in healthy individuals. Another finding of the study was that the degree of swallowing anxiety in the PD group was higher than that in healthy individuals.

Anatomic and/or biomechanical differences can cause swallowing difficulty. In particular, physiological changes can increase the likelihood that an individual experiences PD [22]. One of these physiological differences can be a dysfunction that may occur in the muscles that function during swallowing. Functional swallowing is a complex process that occurs with simultaneous contractions of the muscles responsible for swallowing [31]. A negative difference at any stage of this process can cause swallowing difficulty, directly affecting swallowing function. A decrease in hyoid bone movement or larynx elevation is accepted as one of the most important causes of a swallowing disorder [32]. Once the SH muscles activate, the hyoid bone and larynx rise, causing the bolus to pass into the esophagus [33]. Therefore, investigating the physiological and biomechanical properties of the SH muscles during functional swallowing is important for identifying the problem and planning the rehabilitation process [34]. As a result of the study, comparisons were made between the thicknesses of the anterior venter section of the digastric muscle, mylohyoid muscle, and geniohyoid muscle, which constitute the SH muscles, and the extent of muscle contraction in healthy individuals with and without PD. In the present study, the thickness values of all three SH muscles were lower in healthy individuals with PD than in those without PD, in the resting position. It has been shown that, in particular, a decrease in the strength of the SH muscles negatively affects swallowing function by causing weakness in hyoid movement [35]. An increase in swallowing muscle strength occurs in parallel with the increase in SH muscle thickness [36]. A decrease in muscle thickness can lead to reduced muscle strength, resulting in difficulty swallowing [37,38]. Conversely, an increase in strength following SH muscle strength training has been shown to have positive effects on symptoms of swallowing difficulty and water-swallowing performance [39]. In healthy individuals with PD, the decrease in muscle thickness can also be considered a decrease in muscle strength, and this may have contributed to the development of PD. However, in this study, muscle thickness was measured in the resting position. Measuring muscle thickness during a contraction when swallowing will also allow more accurate results to be obtained and interpreted. The main causes of swallowing difficulties may include aging and neurological problems. However, none of the individuals participating in this study had a neurological diagnosis or neurological findings, and their ages ranged from 18 to 40 years. In addition, researchers did not identify any clinical complaints or symptoms during the study that could have caused a decrease in muscle thickness. According to Yamamoto et al., it was reported that while individuals with PD had no difficulty in swallowing food, they experienced difficulty when swallowing different types of pills. There was an increase in the number of swallows and sEMG explosive muscle activity when swallowing pills in the study [40]. It is thought that there is a need for randomized controlled trials that specifically examine the effects of SH muscle training in healthy individuals with PD.

The Water-Swallowing Test (TWST) is a widely used objective clinical assessment tool to screen patients for dysphagia or aspiration risk [41,42]. Objective quantitative measurement in the assessment of dysphagia can improve the accuracy of screening and serve as a metric indicator of the recovery process. Low swallowing capacity measured in TWST can indicate poor swallowing function [43]. In the present study, the water-swallowing time with/without pills was longer, and the water-swallowing speed and the water-swallowing capacity were also lower in the PD group. However, there was no difference between the two groups with respect to the water volume swallowed in each swallow. In this study, the reduced muscle thickness observed in individuals with PD may have resulted in insufficient muscle activity necessary for water swallowing.

In this study, the difficulty experienced by healthy individuals with dysphagia (PD) when swallowing pills was evaluated using the VAS (Visual Analog Scale). The mean VAS score for individuals in the PD group was 8.90 ± 1.29 (range, 6.00–10.00), while the mean VAS score for individuals in the control group was 0.20 ± 0.41 (range, 0.00–1.00). This difference clearly demonstrates the greater difficulty healthy individuals with PD experience when swallowing pills. It is thought that the negative differences in muscle thickness in individuals with PD may lead to difficulty swallowing pills because they cannot generate sufficient muscle activity for swallowing.

Swallowing anxiety (SA) can be defined as “fear of or avoiding swallowing food, liquids, and pills”, and is also known as a swallowing phobia in the literature [44]. In a study of older adults, a close relationship between swallowing difficulty and SA was observed, and it was stated that detecting swallowing difficulty and starting rehabilitation early could be beneficial for SA [45]. The SAS was used in the current study, and significant differences were determined between the groups. The points on the questionnaire evaluating swallowing anxiety were observed to be higher in the PD group. These findings suggest that healthcare professionals working in this field should consider the possibility that anxiety levels may be high when communicating with healthy individuals who have PD.

This study had some limitations, primarily that participants were not questioned about when and how PD emerged. It may be essential to investigate the qualitative data that could affect the development of PD. Another significant limitation is that the suprahyoid (SH) muscle thickness was measured at rest. It is also necessary to examine whether there are differences in the SH muscle thickness during swallowing. Another limitation was that the reasons for PD could vary in different age groups, and thus comparisons according to age groups could shed light on identifying and solving this problem. However, this study was conducted with young individuals, controlling for the negative effects of anatomical and physiological changes that may occur with aging. Nevertheless, similar assessments and results need to be presented in elderly individuals with PD.

## 5. Conclusions

Pill dysphagia (PD) is defined as a significant problem that compromises individual and public health by leading to medication non-adherence and unauthorized modifications of solid oral dosage forms. In healthy individuals with PD, a distinct decrease in SH muscle thickness and water-swallowing performance, alongside an increase in swallowing anxiety, was observed. Considering the critical importance of the SH muscles in the swallowing mechanism, it is hypothesized that the reduction in SH muscle thickness may be associated with the difficulty or inability to swallow pills experienced by these otherwise healthy individuals. The literature does not describe a definitive treatment method for these patients; instead, it focuses on techniques aimed at making it easier to swallow pills. Consequently, findings from studies investigating the effects of SH muscle training in healthy individuals with PD may guide future therapeutic interventions.

## Figures and Tables

**Figure 1:**
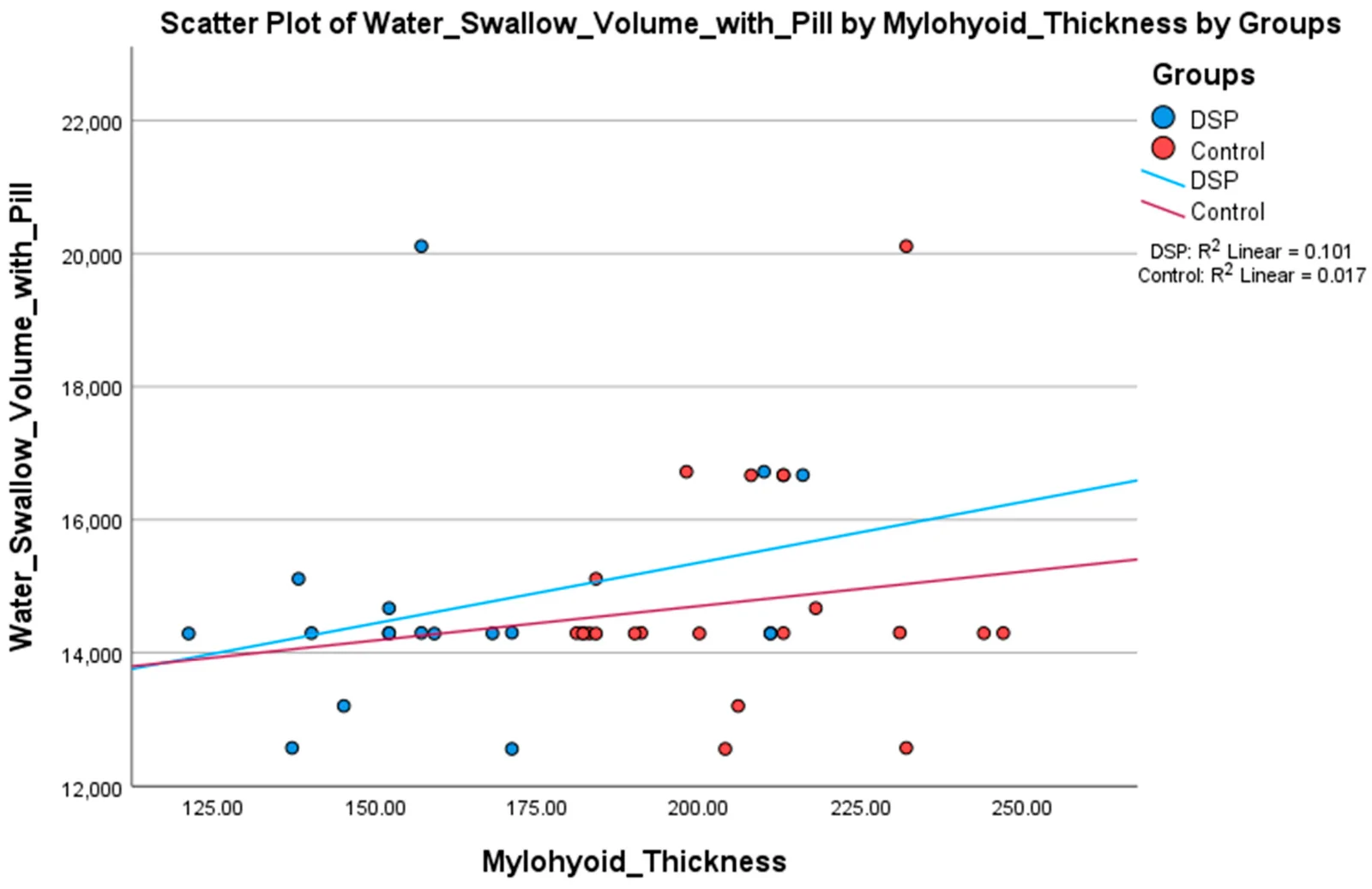
Scatter plot of water swallow volume with pills by mylohyoid thickness in groups.

**Table 1:** Comparisons of the demographic data of the groups.

	Group		N	Mean ± SD	*p*
Age (years)	Control		20	24.25 ± 2.36	0.565 **
	PD		20	23.70 ± 3.53	
Weight (kg)	Control		20	74.30 ± 8.44	0.646 **
	PD		20	71.65 ± 24.19	
Height (cm)	Control		20	177.75 ± 8.01	0.112 **
	PD		20	172.20 ± 9.87	
BMI	Control		20	23.42 ± 0.91	0.134 **
	PD		20	22.30 ± 2.54	
Gender	Control	Female	16		
		Male	4	-	0.176 ^¥^
	PD	Female	11		
		Male	9		

PD: Difficulty in Swallowing Pills; SD: Standard Deviation; BMI: Body Mass Index; **: Independent Samples *t*-test; ^¥^: Chi-Square Test; *p* < 0.05.

**Table 2:** Comparison results of evaluation parameters between groups.

	Group	Mean ± SD	Min–Max	*p*
Digastric Anterior Venter Muscle Thickness (mm)	Control (n:20)	8.14 ± 1.27	6.6–11.6	0.000 **
	PD (n:20)	6.69 ± 1.03	5.0–8.2	
Mylohyoid Muscle Thickness (mm)	Control (n:20)	20.71 ± 2.13	18.1–24.7	0.000 **
	PD (n:20)	16.82 ± 2.94	12.1–21.6	
Geniohyoid Muscle Thickness (mm)	Control (n:20)	9.17 ± 0.89	8.1–11.4	0.007 **
	PD (n:20)	7.90 ± 1.80	5.3–10.5	
Water Swallow Speed (secs/number of swallows)	Control (n:20)	1.51 ± 0.27	1.09–2.07	**0.005 ^++^**
	PD (n:20)	1.72 ± 0.17	1.48–2.05	
Water Swallow Capacity (100 mL/s)	Control (n:20)	8.99 ± 0.63	8.07–10.13	**0.000 ^++^**
	PD (n:20)	7.66 ± 0.62	6.65–9.07	
Water Swallow Volume (100 mL/number of swallows)	Control (n:20)	13.58 ± 1.84	10–16.67	0.468 ^++^
	PD (n:20)	13.16 ± 1.12	11.11–14.29	
Water Swallow Speed with Pill (secs/number of swallows)	Control (n:20)	1.61 ± 0.18	1.34–2.04	**0.000 ^++^**
	PD (n:20)	2.04 ± 0.31	1.43–2.61	
Water Swallow Capacity with Pill (100 mL/s)	Control (n:20)	8.66 ± 0.52	7.77–9.52	**0.000 ^++^**
	PD (n:20)	7.07 ± 0.53	6.22–8.05	
Water Swallow Volume with Pill (100 mL/number of swallows)	Control (n:20)	13.9 ± 1.27	12.5–16.67	0.162 ^++^
	PD (n:20)	14.52 ± 1.45	12.5–16.67	
EAT-10 (points)	Control (n:20)	0.00 ± 0.00	0.00–0.000	**0.001 ^++^**
	PD (n:20)	3.50 ± 1.55	2.00–4.00	
Swallowing Anxiety Scale (points)	Control (n:20)	0.00 ± 0.00	0.00–0.00	**0.001 ^++^**
	PD (n:20)	7.50 ± 1.79	5.00–12.00	
Degree of Difficulty Felt when Swallowing a Pill (cm)	Control (n:20)	0.20 ± 0.41	0.00–1.00	0.000 **
	PD (n:20)	8.90 ± 1.29	6.00–10.00	

PD: Difficulty in Swallowing Pills; SS: Standard Deviation; ^++^: Mann–Whitney U-test; **: Independent Samples *t*-test; *p* < 0.05.

**Table 3:** ANCOVA results for suprahyoid muscle thicknesses by group, controlling for sex and BMI.

Dependent Variable (Muscle)	Covariate/Factor	*df*	*F*	*p*	*η* ^2^
Digastric Muscle Thickness	Covariate (Sex)	1	16.30	<0.001	0.31
	Group	1	11.79	0.001	0.24
	Covariate (BMI)	1	5.73	0.022	0.13
	Group	1	11.10	0.002	0.23
Mylohyoid Muscle Thickness	Covariate (Sex)	1	9.75	0.003	0.21
	Group	1	17.73	<0.001	0.32
	Covariate (BMI)	1	3.00	0.092	0.08
	Group	1	17.73	<0.001	0.32
Geniohyoid Muscle Thickness	Covariate (Sex)	1	11.13	0.002	0.23
	Group	1	4.63	0.038	0.11
	Covariate (BMI)	1	7.43	0.010	0.17
	Group	1	4.63	0.038	0.11

df: Degrees of Freedom; η^2^: Partial Eta Squared. For all models, error degrees of freedom (df) = 37, and total degrees of freedom (df) = 40.

## Data Availability

The data that support the findings of this study are available on request from the corresponding author. The data are not publicly available due to privacy or ethical restrictions.

## Abbreviations

The following abbreviations are used in this manuscript:

PD	Pill Dysphagia
SH	Suprahyoid
SAS	Swallowing Anxiety Scale
SA	Swallowing Anxiety
SD	Swallowing Disorder
WST	Water-Swallowing Test
VAS	Visual Analog Scale
